# Adenosine Deaminase Levels in CSF of Tuberculous Meningitis Patients

**DOI:** 10.4021/jocmr429w

**Published:** 2010-10-11

**Authors:** Bharat Kumar Gupta, Anchit Bharat, Bandyopadhyay Debapriya, Haren Baruah

**Affiliations:** a Department of Biochemistry, Subharti Medical College, S. V. S. University, Meerut- 250005, India; b Medical College, S. V. S. University, Meerut-250005, India

## Abstract

**Background:**

Tuberculosis kills five lakh patients in India every year, out of which 7-12 % are with meningeal involvement. Delay in its diagnosis and in initiation of treatment results in poor prognosis and sequlae in up to 25% of cases. The aim of the present study is to look for a simple, rapid, cost effective, non-invasive and fairly specific test in differentiating tubercular etiology from other causes.

**Methods:**

Forty patients between the age of 6 - 24 months attending hospital with symptoms and signs of meningitis were selected and divided into two groups: tubercular and non-tubercular, depending upon the accepted criteria. CSF was drawn and ADA estimated.

**Results:**

Out of 19 tubercular patients, 18 had CSF ADA at or above the cutoff value while one had below. Out of 21 non-tuberculous patients, two had ADA levels at or above the cutoff value while 19 had below this value. Results of our study indicate that ADA level estimation in CSF is not only of considerable value in the diagnosis of TBM, CSF ADA level 10 U/L as a cutoff value exhibited 94.73% sensitivity and 90.47% specificity in differentiating tuberculous from non-tuberculous meningitis; it also has 90.00% positive predictive value and 95.00% negative predictive value.

**Conclusions:**

It can be concluded that ADA estimation in CSF is not only simple, inexpensive and rapid but also fairly specific method for making a diagnosis of tuberculous etiology in TBM, especially when there is a dilemma of differentiating the tuberculous etiology from non-tuberculous ones. For this reason ADA estimation in TBM may find a place as a routine investigation.

**Keywords:**

Cerebrospinal fluid; Adenosine deaminase; Tuberculous meningitis

## Introduction

Tuberculous meningitis (TBM) is an endemic disease in developing countries [1], more so in low socio-economic status. Five lakh patients of tuberculosis die every year in India [2], 8.3 % of which is childhood tuberculosis. Multidrug resistance in tuberculosis and acquired immuno-deficiency syndrome (AIDS) further worsen the outcome of this disease [3]. Incidence of TBM in developing countries is 7-12%. Delay in diagnosis and so in the start of effective treatment results in poor prognosis and sequalae in up to 25% of cases [4]. Available methods of diagnosis of TBM were evaluated [5] and all of them were found to have low sensitivity and specificity.

The newer methods for diagnosing tubercular disease is based on pheno- and genotypic methods. For the detection of acid fast bacilli (AFB) in a smear, light microscopy is a common, rapid and specific method and is used the world over with a detection rate of 30-40% [6]. Sensitivity of culture on Lowenstein-Jensen (L-J) medium is higher than microscopy but it needs several weeks of incubation. A number of genotypic assays based on nucleic acid amplification have been designed including GenProbe amplified Mycobacterium tuberculosis direct test, Roche Amplicor MTB test, Cobas Amplicor test, Abbott LCx test, and the BD-Probe Tec (strand displacement amplification) test [7-11]. However, high cost involved in these tests prevents them to be widely used especially in developing countries.

Adenosine deaminase (ADA) is an enzyme in the purine salvage pathway that catalyzes the conversion of adenosine and deoxyadenosine to inosine and deoxyinosine respectively with the release of ammonia. It plays important role in differentiating lymphoid cells and is present in abundance in active T-lymphocytes whose concentration is inversely proportional to the degree of differentiation [12]. Its levels are ten times higher in T-lymphocytes than in erythrocytes. The enzyme activity increases during mitogenic and antigenic responses of lymphocytes and T-lymphocyte blastogenesis can be inhibited by inhibitors of ADA. Likewise, a deficiency of adenosine deaminase is associated with severe defects in the cell mediated and the humoral arms of the immune system, predisposing the patient to opportunistic infections.

ADA is released by T cells during cell mediated immune response (CMI) to the tubercle bacilli. ADA is now being recognized as a marker of cell mediated immunity particularly as a marker of T lymphocyte activation. Adenosine deaminase levels (ADA) have also been considered by several researchers to differentiate tubercular disease from non-tubercular [13-17].

Different modalities to support the diagnosis in cases of TBM have to be considered because the cytological and biochemical analysis of cerebro-spinal fluid (CSF) have a considerable overlap. As few studies have been conducted to evaluate the role of CSF ADA levels for the diagnosis of TBM, we tried to estimate ADA levels in CSF in TBM and to find out its role as a sensitive, accurate, rapid, and affordable diagnostic tool that will work in resource-limited settings in confirming the tubercular etiology in cases of meningitis.

## Patients and Methods

Routine procedures of history taking, examination and routine investigations were followed. Forty patients between the age of six months to two years having symptoms and signs of meningitis, admitted in the Pediatrics ward from July 2009 to June 2010 at the Subharti Medical College, Meerut, India were included in the present study.

Presence of first or more than one of the following criteria was adopted to label a case as tuberculous:

1. Bacteriological proof of presence of Mycobacterium tuberculosis.
2. Biopsy showing caseating granulomas.
3. Clinico- radiological findings consistent with TB.
4. Definite clinical and radiological improvement in one month after specific anti-tubercular treatment.
5. History of contact with current disease and positive reaction (> 20 mm induration) to 5 tuberculin unit (TU) purified protein derivative (PPD).

Lumbar puncture was done in each case and at least 2 ml of CSF was collected in a sterile vial. Hemorrhagic CSF was excluded from the study. This CSF was subjected to biochemical and microscopic examination. ADA activity was estimated in all these patients by the method of Guisti [18] and was expressed as U/L. Cutoff reference range of 10 U/L CSF ADA was taken as positive.

## Results

Out of 40 patients, 28 children were male and 12 patients were female (Table 1). Nineteen patients (47.5%) having fulfilled the criteria were labeled as tubercular, while the other 21 (52.5%) were labeled as non-tubercular (Table 2). Only two (10.52%) patients in 19 tubercular had evidence of presence of Mycobacterium.

**Table 1:** Distribution of the Cases According to Sex

Sex	Number of cases
Male	28
Female	12
Total	40

**Table 2:** Distribution of the Cases According to Accepted Criteria

Group	Number of cases
Tuberculous	19 (47.5%)
Non-tuberculous	21 (52.5%)
Total	40

Out of these 19 tuberculous patients, 18 patients were found to be having CSF ADA at or above the cutoff value while only one had below cutoff value. Of the 21 patients labeled non-tuberculous, two patients were found to be having CSF ADA at or above the cutoff value while 19 had values below the cutoff (Table 3).

**Table 3:** Distribution of the Cases According to Set Criteria and CSF ADA Levels

Group	Number of cases	ADA levels in U/L		Mean SD	t cal	P-value
Tuberculous	19	ADA ≥ 10	18	27.1684		
		ADA < 10	01	22.7660		
Non-tuberculous	21	ADA ≥ 10	02	6.0619	4.0173	0.0007
		ADA < 10	19	2.5714		(p < .01)*
Total	40		40			

*(p < 0.01) shows a highly significant difference between the tuberculous and non-tuberculous groups.

In tuberculous group ADA activity in CSF ranged between 9.2 to 110 U/L with a median of 22, mean SD as 27.1684 (ADA ≥ 10) and 22.7660 (ADA < 10). While in non-tuberculous group ADA activity ranged between 2 to 10.5 U/L with a median of 6, mean SD as 6.0619 (ADA ≥ 10) and 2.5714 (ADA < 10).

On comparison of the values of CSF ADA in the two groups, t cal is 4.0173 and the difference in these two values was found highly significant (p < 0.01) (Table 3).

## Discussion

Demonstration of AFB in CSF, CSF culture, CSF cytology, and genomic amplification are the various means to confirm the etiology of TBM but visualization of AFB in direct smears or in cultures of CSF is usually difficult [19] and in most cases negative. Newer methods such as those involving bacterial genomic amplifications by PCR or other comparable methods, are not available for widespread use in the developing countries.

Routine CSF laboratory findings may not be helpful to differentiate tuberculous etiology in meningitis from other causes. The aim is to look for a simple and rapid test that can help in the diagnosis of TBM and differentiate it from other causes with fairly good accuracy.

ADA has been considered as a marker of cell-mediated immunity and its activity has been observed in various infections including TBM. Considering that both humoral and cell-mediated immunity play an important role in TBM infection, it has been suggested that ADA activity in CSF may help differentiate TBM from non-TBM infectious meningitis.

CSF - ADA estimation was reported to be useful in diagnosing TBM and to differentiate TBM from normal subject or patients with other neurological disorders [20]. CSF - ADA estimation is a useful method to differentiate TBM from aseptic meningitis [13]. Other researchers have also observed the usefulness of CSF-ADA activity in the diagnosis of TBM [21, 22].

In the present study CSF - ADA level 10 U/L as a cut-off value differentiate tuberculous from non-tuberculous meningitis. We have observed a highly statistically significant difference in the CSF - ADA levels of meningitis due to tuberculosis and non-tuberculous etiology (P < .01) (Table 3). Results of our study indicate that ADA levels in CSF are of considerable value in diagnosis of TBM and in differentiating this disease from others because a cut-off CSF - ADA level of 10 U/L exhibited fairly high accuracy with sensitivity of 94.73%, specificity of 90.47% for the diagnosis of tuberculous meningitis. In addition to this, the positive predictive value of test is 90.00% with overall accuracy being 95.00% (Table 4). In cases where there is a dilemma in confirming the etiology, CSF ADA levels help in reaching out to a conclusion, and depending upon the values of ADA, patient may be diagnosed and treated.

**Table 4:** Distribution of the Cases According to True and False Positivity and Negativity and Statistical Accuracy

Cell Entries	Number of Cases	Statistical Indices of Diagnostic Accuracy	Percentage
True Positive	18	Sensitivity	94.73%
False Negative	01	Specificity	90.47%
True Negative	19	Positive predictive valve	90.00 %
False Positive	02	Negative predictive value	95.00%

Levels of ADA in CSF of adult patients of TBM have been evaluated in earlier studies [12-14]. Raised levels of ADA in CSF are not specific to meningeal inflammatory disease but it can be a test for confirming its etiology with good predictive value. Raised ADA levels have also been noted in other conditions particularly in certain intracranial tumors [15].

Gupta et al [16] observed that adenosine deaminase levels in nontuberculous disease rarely exceeded the cut-off; set for tuberculous disease. They [17] have further observed that ADA estimation is not only a fairly sensitive and specific test (more than 90 %), helpful in differentiating tubercular from non-tubercular etiology; both in pulmonary and extra-pulmonary disease but is also simple, inexpensive and rapid. For this reason this test may help in early diagnosis, improve the prognosis and reduce spread of disease and sequlae.

The mean ADA levels in CSF of TBM cases of pediatric age groups have been reported to be ranging between 11.6-13.7 U/L in earlier study [12]. A level 15.7 - 21.3 U/L has been observed in adult TBM patients [13, 14]. These results show that levels of ADA vary in different age groups. In our study CSF ADA levels were found to be 9.2 to 110 U/L in tuberculous group with a median of 22, mean SD as 27.1684 (ADA ≥ 10) and 22.7660 (ADA < 10); while in non-tuberculous group ADA activity ranged between 2 to 10.5 U/L with a median of 6, mean SD as 6.0619 (ADA ≥ 10) and 2.5714 (ADA < 10). All the patients were between the age group of 6 to 24 months. This may be due to difference in immunological reactivity to tubercular antigen in children as compared to adults.

Kashyap et al take cutoff value of 11.39 U/L and has obtained sensitivity of 82% and specificity as 83% in TBM cases [15]. Rana et al take 10 U/L as cutoff value for diagnosis of TBM and found sensitivity 66.6% and specificity 90% [23]. Baheti et al found that CSF ADA may differentiate tuberculous from non-tuberculous meningitis even at a cut-off level of 6.5 U/L [24].

In the present study, median ADA levels in CSF were significantly high in TBM patients as compared to those with other etiologies. Ribera et al have also demonstrated similar finding but his study was in TBM patients of adult age group [25].

In conclusion, we found the sensitivity of the test to be 94.73%; specificity 90.47%, positive predictive value is 90.00 % and negative predictive value 95.00%, and so it can be concluded that ADA estimation in CSF is not only simple, inexpensive and rapid but also fairly specific method for making a diagnosis of tuberculous etiology in TBM, especially when there is a dilemma of differentiating the tuberculous etiology from non-tuberculous. For this reason CSF ADA estimation in TBM may find a place as a routine investigation.
